# Quality-of-life evaluation for advanced non-small-cell lung cancer: a comparison between vinorelbine plus gemcitabine followed by docetaxel versus paclitaxel plus carboplatin regimens in a randomized trial: Japan Multinational Trial Organization LC00-03 (BRI LC03-01)

**DOI:** 10.1186/1471-2407-11-356

**Published:** 2011-08-17

**Authors:** Masaaki Kawahara, Harue Tada, Akihiro Tokoro, Satoshi Teramukai, Hideki Origasa, Kaoru Kubota, Tetsu Shinkai, Masanori Fukushima, Kiyoyuki Furuse

**Affiliations:** 1 Otemae Hospital, 1-5-3 Otemae, Chuo-ku, Osaka, 540-0008, Japan; 2 Department of Clinical Trial Design and Management, Translational Research Center, Kyoto University Hospital, 54 Shogoin Kawahara-cho, Sakyo-ku, Kyoto, 606-8507, Japan; 3 National Hospital Organization, Kinki-chuo Chest Medical Center, 1180 Nagasone-cho, Kita-ku, Sakai, 591-8555, Japan; 4 Department of Biostatistics and Clinical Epidemiology, University of Toyama Graduate School, 2630 Sugitani, Toyama, 930-0194, Japan; 5 National Cancer Center Hospital, 5-1-1 Tsukiji, Chuo-ku, Tokyo, 104-0045, Japan; 6 National Hospital Organization Shikoku Cancer Center, 160 Minami Umemoto-cho, Matsuyama, 791-0280, Japan; 7 Translational Research Informatics Center, 1-5-4 Minatojima-minamimachi, Chuo-ku, Kobe, 650-0047, Japan; 8 The Japan Multinational Trial Organization, 474 Uehonnojimae-cho, Teramachi-Oike agaru, Nakagyo-ku, Kyoto, 604-0925, Japan

## Abstract

**Background:**

A randomized trial of vinorelbine plus gemcitabine followed by docetaxel (VGD) versus paclitaxel plus carboplatin (PC) in patients with advanced non-small-cell lung cancer showed no difference in overall survival (median survival time: 13.6 versus 14.1 months) between the two treatment groups. We report here the results of quality-of-life (QOL) study initiated in the mid-course of this randomized trial.

**Methods:**

The patients themselves assessed the Functional Assessment of Cancer Therapy (FACT)-Lung (FACT-L), FACT-Taxane and the Functional Assessment of Chronic Illness Therapy - Spirituality (FACIT-Sp) QOL instruments at baseline and 6, 12 and 18 weeks after the treatment. The primary endpoint was a comparison of total QOL score for each assessment instrument between the two groups.

**Results:**

Sixty-eight patients from the trial (VGD, 34; PC, 34) who submitted baseline questionnaires and at least one questionnaire over the course of treatment were eligible. Longitudinal analysis showed a significant difference in slope of the FACT-Taxane score (p = 0.004) between treatment regimens over time, but no difference was found in FACT-L score (p = 0.311) and FACIT-Sp score (p = 0.466) between the two groups.

**Conclusions:**

The significant difference in slope of FACT-Taxane score favored the VGD regimen. These data should be considered in treatment decision-making for patients with advanced non-small-cell lung cancer.

**Trial registration:**

NCT00242983.

## Background

Non-small-cell lung cancer (NSCLC) accounts for approximately 75-85% of all cases of lung cancer [1-3]. More than 70% of patients have locally advanced or incurable metastatic disease [4]. Overall 5-year survival is approximately 14%. The goal of treatment for patients with recurrent or metastatic NSCLC remains palliative. Experience over the past three decades has shown improvements in survival, symptom control and quality of life (QOL) in patients with metastatic NSCLC who receive first-line chemotherapy [5-7]. The benefits of treatment must outweigh the risks, and patient-focused outcomes and optimization of well-being are of paramount importance for individuals who have limited life expectancy [8,9]. Thus, we designed a QOL study (BRI LC03-01) to be run during an ongoing randomized controlled trial (Japan Multinational Trial Organization (JMTO) LC00-03[10]). Data were obtained using the Functional Assessment of Cancer Therapy (FACT)-Lung (FACT-L), FACT-Taxane and the Functional Assessment of Chronic Illness Therapy - Spirituality (FACIT-Sp) QOL assessment instruments.

## Methods

### Study population

**Figure 1:**
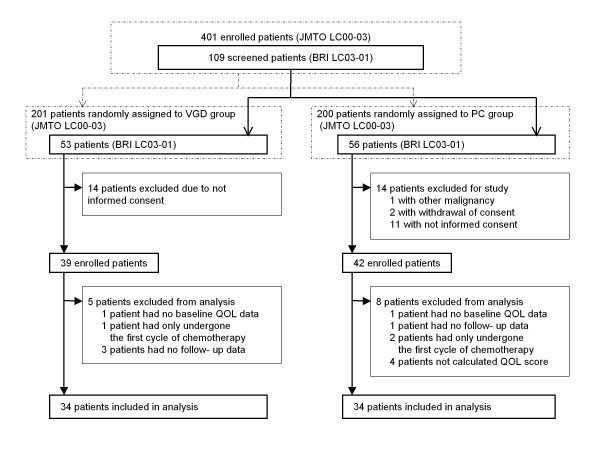
**Trial profile**.

This study (BRI LC03-01) was conducted as one of the additional studies of JMTO LC00-03 trial. As an exploratory trial, this study was started in the mid-course of the JMTO LC00-03 trial period (Figure 1). The comprehensive details on the whole patient population studied and methods employed was shown in the JMTO LC00-03 report [10]. Briefly, between March 2001 and April 2005 from 45 institutions in Japan, 401 chemotherapy-naïve NSCLC patients with stage IIIB disease with pleural effusion or stage IV disease without brain metastasis were randomized to one of two treatment regimens. These were either the vinorelbine plus gemcitabine followed by docetaxel (VGD) group (intravenous vinorelbine 25 mg/m^2 ^plus gemcitabine 1000 mg/m^2 ^on days 1 and 8 every 21 days for three cycles, followed by intravenous docetaxel 60 mg/m^2^, on day 1 every 21 days for three cycles) or the paclitaxel plus carboplatin (PC) group (intravenous paclitaxel 225 mg/m^2 ^plus carboplatin AUC = 6 for 3 h on day 1 every 21 days for six cycles). The eligibility criteria for this study (BRI LC03-01) were the same as for the JMTO LC00-03 trial and included written informed consent to enter this QOL study. The criteria of the JMTO LC00-03 trial included cytologically or histologically confirmed NSCLC, stage IIIB (plueral effusion) or IV, chemotherapy-naïve, age 18 years and over, ECOG performance status 0 or 1, adequate organ function and no brain metastasis. The study protocol was approved by the ethics committee at each institution.

### QOL assessment

QOL was assessed using FACT-L [11], FACT-Taxane [12], and FACIT-Sp [13,14] at baseline (pre-treatment), prior to the third treatment cycle (Week 6), prior to the fifth treatment cycle (Week 12), and at the end of sixth treatment cycle (Week 18) or at study withdrawal. In this study, these were self-administered by patients.

#### FACT-L

The FACT-L instrument is a multidimensional questionnaire developed and validated for use in lung cancer patients; it includes the 27-item FACT-General (FACT-G; version 4 in Japanese) questionnaire targeted to general cancer patients and seven questions specific to issues faced by lung cancer patients (lung cancer subscale, LCS). The FACT-G version 4 questionnaire includes the following four subscales: physical well-being (PWB; seven items), social/family well-being (seven items), emotional well-being (six items) and functional well-being (FWB; seven items). These subscales can be analyzed separately or aggregated to produce a total QOL score. FACT-G has demonstrated reliability, validity and responsiveness to change over time [15].

#### FACT-Taxane

FACT-Taxane comprises the FACT-G questionnaire plus a 16-item Taxane subscale (version 4 in Japanese). The Taxane subscale combines the validated 11-item neurotoxity subscale and five additional questions assessing symptoms related to arthralgia, myalgia and skin discoloration. The FACT-Taxane instrument is validated for use in lung cancer patients [12].

#### FACIT-Sp

FACIT-Sp comprises the FACT-G questionnaire plus a 12-item spirituality subscale (version 4 in Japanese). The FACIT-Sp subscale comprises two factors. One of these (labeled meaning/peace) includes eight items and assesses the sense of meaning, peace and purpose in life. The other (labeled faith) contains four items and measures several aspects of the relationship between illness and one's faith and spiritual beliefs. The Japanese version of the FACIT-Sp scale is satisfactory in terms of reliability and validity and is a useful tool in the study of spirituality among Japanese cancer patients including lung cancer [14].

FACT-G, lung cancer subscale, Taxane subscale and spirituality subscale were each scored using a five-point scale (0 = not at all; 1 = a little bit, 2 = somewhat; 3 = quite a bit; 4 = very much). According to Functional Assessment of Chronic Illness Therapy (FACIT) scoring guidelines [15], these scores were calculated only when patients responded to at least 80% of the items that constituted the relevant score. The ranges of possible total scores are 0-136 in FACT-L, 0-172 in FACT-Taxane and 0-156 in FACIT-Sp. The FACT-G score is calculated by summing four of the five unweighted subscale scores, specially the PWB, SWB, EWB, and FWB scores (i.e., excluding the LCS), with score in the range of 0-108. The trial outcome index (TOI), an efficient summary index of physical/functional outcomes, is the sum of the PWB, FWB, and specific subscale (LCS or Taxane subscale) scores. The ranges of TOI scores are 0-84 in FACT-L and 0-120 in FACT-Taxane. For all items and domains, higher scores are associated with better QOL.

### Statistical considerations

The primary objective of this study was to test whether the VGD regimen produced better QOL compared with the PC regimen in patients with advanced NSCLC. The primary endpoints were total scale scores of FACT-L, FACT-Taxane, and FACIT-Sp. Secondary endpoints were subscale scores of FACT-L, FACT-Taxane, and FACIT-Sp. We initially planned that we could enrol 200 patients on the basis of the feasibility.

The mean scores for the specific scales were calculated within groups based on treatment at each period of the assessment. A change in QOL outcome was calculated by subtracting an individual's scores during treatment from that patient's baseline score. Patients who dropped out and only submitted a QOL assessment at the baseline were excluded from this study. We assumed that missing a QOL assessment was a random event (missing at random, MAR): that discontinuation of the QOL assessment depended on previous QOL assessments, but was independent of future ones. QOL assessments in a group-based analysis were performed by analyzing changes of the mean scores over the course of treatment with a general linear mixed-effects model. The mixed-effects modeling included the main effect of treatment group (difference in overall mean between groups) and an interaction effect of treatment group and time (difference in slope between groups) under the MAR assumption. Statistical analyses were performed by using SAS version 9.1 (SAS Institute, Cary, NC, USA).

## Results

Out of 401 enrolled patients in JMTO LC00-03, 109 patients were screened for BRI LC03-01 between January 2004, and April 2005, from 14 institutions in Japan (Figure 1). Twenty-five patients (VGD group, 14; PC group, 11) who did not provide the consent for the QOL study, and three in PC group were ineligible for the JMTO LC00-03 trial (1 had other malignancy and 2 withdrew informed consent). Although total eighty-one patients were enrolled in this study, thirteen patients (VGD group, 5; PC group, 8) were excluded from the analysis: two patients had no baseline QOL data (VGD group, 1; PC group, 1), four patients had no QOL follow-up data (VGD group, 3; PC group, 1), three patients had only undergone the first course of chemotherapy (VGD group, 1; PC group, 2), and four patients fulfilled QOL in less than 80% of any one questionnaire (FACT L, FACT-Taxane, and FACIT-Sp) at baseline (two patients) and after the second course of chemotherapy (two patients) in PC group. Sixty-eight patients from the trial (VGD group, 34; PC group, 34), who submitted both a baseline questionnaire and at least one questionnaire over the course of the treatment, were included in the analysis.

Table 1 shows the patient characteristics of each group. Significantly more female patients were distributed to the PC group (44%) than to the VGD group (18%) (p = 0.003). Other factors such as age, ECOG performance status, histology, stage, weight loss and serum lactate dehydrogenase (LDH) were well balanced between the PC and VGD groups. More than half of the patients had adenocarcinoma and/or stage IV disease.

**Table 1:** Baseline patients characteristics

Characteristics	PC group (n = 34)	VGD group (n = 34)
Age, years		
Median	66	64
Range	(33-75)	(39-79)
Sex, n (%)		
Male	19 (56%)	28 (82%)
Female	15 (44%)	6 (18%)
ECOG performance status, n (%)		
0	12 (35%)	15 (44%)
1	22 (65%)	19 (56%)
Histologic type, n (%)		
Adenocarcinoma	26 (76%)	22 (65%)
Squamous cell	5 (15%)	9 (26%)
Other	3 (9%)	3 (9%)
Stage, n (%)		
IIIB	3 (9%)	4 (12%)
IV	31 (91%)	30 (88%)
Weight loss (from 6 months before enrolment, n (%)		
<5%	29 (85%)	31 (91%)
≥5%	5 (15%)	3 (9%)
LDH concentration, n (%)		
Normal	28 (82%)	28 (82%)
Abnormal	6 (18%)	6 (18%)

LDH, lactate dehydrogenase.

**Figure 2:**
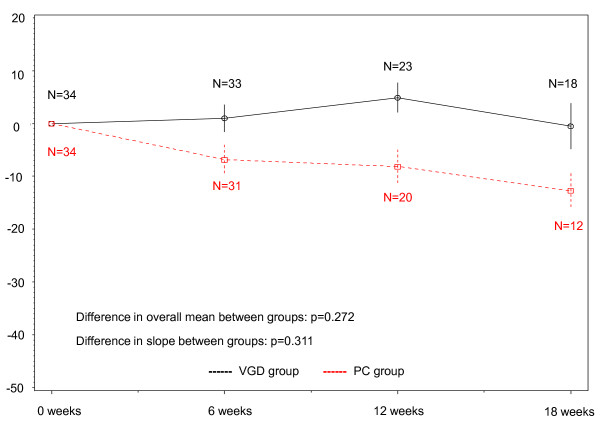
**Changes in FACT-L score**.

**Figure 3:**
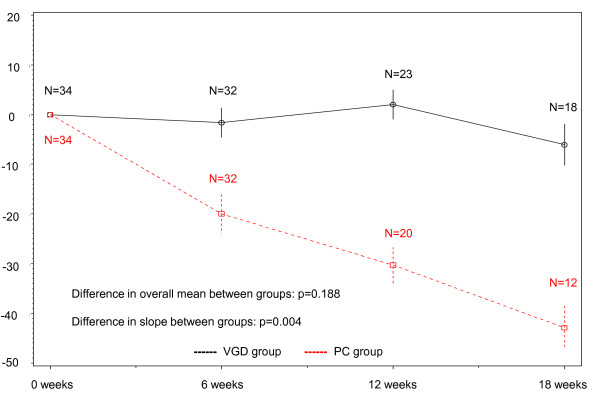
**Changes in FACT-Taxane score**.

**Figure 4:**
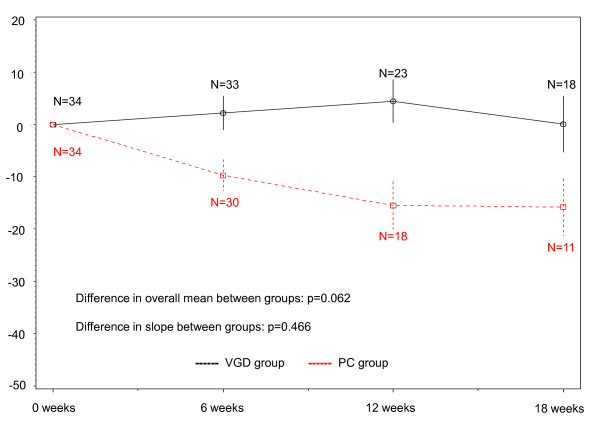
**Changes in FACIT-Sp score**.

Compliance with QOL assessment in each group is shown in Table 2. In both groups, the compliance - rate of submitted questionnaires - was very high; 97% at 6 weeks, 96% at 12 weeks and 92% at 18 week in the PC group and 97, 96 and 90% in the VGD group, respectively. Mean QOL score and subscales in each group are summarized in Table 3. Baseline score showed no significant differences from the overall mean score between groups in the FACT-L (p = 0.272) (Figure 2), FACT-Taxane (p = 0.188) (Figure 3) or FACIT-Sp (p = 0.062) (Figure 4) score. Although there were no significant differences in slope between groups for FACT-L (p = 0.311) (Figure 2) or FACIT-Sp (p = 0.466) (Figure 4), there was a significant difference in slope of the FACT-Taxane score (p = 0.004) (Figure 3). When FACT-Taxane score was assessed over time, the score became significantly worse in the PC group than in the VGD group. We performed post-hoc subgroup analysis according to sex to evaluate the FACT-Taxane score because the VGD group included fewer female patients. The mean changes from baseline score at 18 weeks in the FACT-Taxane instrument for male and female patients were -49.7 (N = 7) and -33.4 (N = 5) in the PC group, and -7.4 (N = 15) and 1.0 (N = 3) in the VGD group respectively. This indicates that there was no difference for change in FACT-Taxane score according to sex between treatment groups.

**Table 2:** Compliance with QOL assessment

	PC group (n = 34)				VGD group (n = 34)			
	
Assessment time	Baseline	6 week	12 week	18 week	Baseline	6 week	12 week	18 week
No. of evaluable patients	34	34	23	13	34	34	24	20

Discontinuation of protocol treatment	0	0	11	10	0	0	10	4

Investigator's error	0	1	1	1	0	1	0	1
No reason provided	0	0	0	0	0	0	1	1

No. of submitted questionnaires Compliance (%)	34 100%	33 97%	22 96%	12 92%	34 100%	33 97%	23 96%	18 90%

**Table 3:** Means QOL scores over time

	PC group (n = 34), mean (SD)				VGD group (n = 34), mean (SD)			
	
Assessment time	Baseline	6 week	12 week	18 week	Baseline	6 week	12 week	18 week
FACT-G								
Physical well-being (7 items)	21.8 (5.3)	16.9 (7.1)	16.1 (5.4)	12.8 (5.7)	21.4 (5.3)	19.1 (6.7)	20.2 (6.2)	20.2 (5.1)
Social/family well-being (7 items)	19.7 (4.7)	19.7 (5.0)	18.9 (6.2)	19.4 (5.2)	18.9 (6.0)	19.6 (4.9)	20.1 (5.1)	17.2 (6.0)
Emotional well-being (6 items)	16.1 (5.1)	15.6 (5.9)	15.5 (6.4)	13.6 (5.6)	14.1 (5.8)	16.3 (5.5)	16.8 (5.1)	16.4 (5.5)
Functional well-being (7 items)	17.5 (6.7)	14.9 (6.3)	15.2 (6.6)	13.1 (5.4)	16.2 (6.9)	17.2 (6.5)	19.2 (6.0)	16.4 (7.2)
FACT-G total (27 items)	73.0 (15.4)	65.1 (17.5)	63.4 (17.4)	57.1 (14.7)	69.0 (12.8)	70.3 (16.7)	74.6 (15.7)	68.9 (16.5)
FACT-L								
Lung cancer subscale (7 items)	19.0 (5.1)	19.3 (3.8)	19.3 (4.8)	18.8 (4.2)	18.9 (3.7)	18.9 (6.0)	20.3 (4.5)	20.4 (5.1)
Trial outcome index-lung (21 items)	57.6 (12.5)	51.2 (13.3)	49.9 (13.8)	44.6 (10.6)	56.2 (10.8)	55.1 (15.9)	59.7 (14.2)	57.0 (14.8)
FACT-Taxane								
Neurotoxicity (11 items)	40.3 (4.1)	30.0 (9.4)	24.3 (9.3)	16.0 (7.2)	39.6 (5.6)	37.4 (7.2)	38.9 (5.5)	37.0 (7.0)
Taxane (16 items)	59.9 (4.3)	47.5 (12.0)	41.0 (11.7)	30.4 (9.9)	59.1 (6.8)	56.5 (8.9)	58.3 (6.1)	55.4 (9.3)
Trial outcome index-taxane (30 items)	98.9 (13.7)	78.9 (21.2)	70.9 (17.4)	55.8 (15.9)	96.7 (11.9)	92.5 (18.0)	97.7 (15.1)	91.8 (16.8)
FACIT-Sp								
Spirituality (12 items)	30.3 (9.8)	30.1 (9.6)	28.3 (11.8)	27.2 (12.4)	28.4 (11.5)	29.6 (9.8)	28.3 (11.4)	30.0 (9.4)

## Discussion

Treatment of advanced metastatic NSCLC is palliative rather than curative and therefore should focus on relief of symptoms as well as extension of survival time. Furthermore, the benefits of chemotherapy should outweigh factors such as toxicity and inconvenience to the patient. Thus, an adequate assessment of the impact of treatment on patient health in the setting of metastatic disease requires measurement of the effects of the therapy on cancer-specific symptoms, treatment-related toxicity and domains of patient health. Assessment of the impact of treatment on QOL provides a comprehensive understanding of the overall burden or benefit of therapy to patients. The present BRI LC03-01 QOL evaluation study demonstrates that patients treated with the VGD regimen generally experienced an improvement in their QOL compared with those in the PC group, but only a statistically significant difference was found in slope of the FACT-Taxane score (p = 0.004). Patients in the VGD group showed no significant mean variation over time in their QOL score, while the patients on the PC group showed a steady decline in FACT-G and FACT-Taxane score over time (Table 3). In terms of general health, patients receiving the VGD regimen had better QOL than those receiving PC treatment, as assessed by the FACT-L, FACT-Taxane and FACIT-Sp scores. JMTO LC00-03, a randomized trial of the VGD and PC regimens in patients with advanced NSCLC, showed differences between the two treatment groups in terms of response rate (25 versus 37%) and regimen-specific toxicity, including taxane-related toxicities such as arthralgia (0 versus 8.6%), myalgia (0 versus 7.1%) and neuropathy (0.5 versus 9.6%). However, there were no differences in overall survival (median survival time, 13.6 versus 14.1 months) [10]. This study of the VGD and PC regimens in patients with advanced NSCLC differences between the two treatment groups in terms of taxane-related toxicities such as grade 3 or 4 arthralgia (0 versus 18%), motor neuropathy (3 versus 12%) and sensory neuropathy (3 versus 18%). The QOL scores of patients with non-small cell lung cancer usually decrease during chemotherapy [16]. However, the QOL score is maintained despite treatment advances in the VGD group. The adverse effect of VGD seems to be less than that of PC. We can be reasonably confident that the statistically significant improvement seen in the QOL of patients treated with either VGD or PC was clinically meaningful, as the results from the QOL assessment tools, in addition to other parameters, such as change in performance status and weight, were very consistent.

The study has some limitations. Firstly, sample size could not be determined based on statistical consideration. Secondly, the numbers of patients actually accrued were fewer than expected, because it took substantial time to get approvement and initiate this trial in each institution in mid-course of the parent phase III trial. Poor compliance with QOL assessments is one of the major methodological problems seen in studies of QOL, as patients experiencing deteriorations in QOL are less likely to comply with future QOL assessments, potentially producing biased results [17]. Although small number, all the patients (PC group: 12, VGD group: 16) who completed the study treatment made the QOL questionnaire completed. This was a well designed and executed randomized clinical trial, but the QOL portion of the trial had these limitation.

## Conclusions

A significant difference in slope of FACT-Taxane was found in favour of non-platinum VGD regimen. Taxane-related toxicities such as arthralgia, myalgia and neuropathy are statistically more frequent in the PC regimen than in the VGD one. Significant differences in other global and specific health-related QOL could not be demonstrated probably due to the limited number of patients. Although this study could be considered a preliminary study, given its limited small sample size, these data, especially FACT-Taxane, should be considered in treatment decision-making for patients with advanced non-small-cell lung cancer.

## List of abbreviations

VGD: vinorelbine plus gemcitabine followed by docetaxel; PC: paclitaxel plus carboplatin; QOL: quality-of-life; FACT-L: Functional Assessment of Cancer Therapy -Lung; FACT-Taxane: Functional Assessment of Cancer Therapy-Taxane; FACIT-Sp: Functional Assessment of Chronic Illness Therapy - Spirituality; NSCLC: non-small-cell lung cancer; JMTO: Japan Multinational Trial Organization.

## Competing interests

The authors declare that they have no competing interests.

## Authors' contributions

MK participated in the design of the study, coordination, and interpretation of data, and drafted the manuscript. HT conceived of the study, participated in the design of the study, performed the data management and the statistical analysis, and drafted the manuscript. AT participated in the design of the study. ST participated in the design of the study, performed the statistical analysis, and helped to draft the manuscript. HO participated in the design of the study and helped drafted the manuscript. KK and TS participated interpretation of data and helped drafted the manuscript. MF participated in the design of the study and helped to draft the manuscript. KF participated in the design. All authors read and approved the final manuscript.

## Pre-publication history

The pre-publication history for this paper can be accessed here:

http://www.biomedcentral.com/1471-2407/11/356/prepub
